# Metagenomic sequencing for detection and identification of the boxwood blight pathogen *Calonectria pseudonaviculata*

**DOI:** 10.1038/s41598-022-05381-x

**Published:** 2022-01-26

**Authors:** Shu Yang, Marcela A. Johnson, Mary Ann Hansen, Elizabeth Bush, Song Li, Boris A. Vinatzer

**Affiliations:** 1 School of Plant and Environmental Sciences, Virginia Tech, Blacksburg, VA USA; 2 Graduate Program in Genetics, Bioinformatics, and Computational Biology, Virginia Tech, Blacksburg, VA USA

**Keywords:** Plant sciences, Fungi, Infectious-disease diagnostics, Pathogens, Computational biology and bioinformatics, Genome informatics

## Abstract

Pathogen detection and identification are key elements in outbreak control of human, animal, and plant diseases. Since many fungal plant pathogens cause similar symptoms, are difficult to distinguish morphologically, and grow slowly in culture, culture-independent, sequence-based diagnostic methods are desirable. Whole genome metagenomic sequencing has emerged as a promising technique because it can potentially detect any pathogen without culturing and without the need for pathogen-specific probes. However, efficient DNA extraction protocols, computational tools, and sequence databases are required. Here we applied metagenomic sequencing with the Oxford Nanopore Technologies MinION to the detection of the fungus *Calonectria pseudonaviculata*, the causal agent of boxwood (*Buxus* spp.) blight disease. Two DNA extraction protocols, several DNA purification kits, and various computational tools were tested. All DNA extraction methods and purification kits provided sufficient quantity and quality of DNA. Several bioinformatics tools for taxonomic identification were found suitable to assign sequencing reads to the pathogen with an extremely low false positive rate. Over 9% of total reads were identified as *C. pseudonaviculata* in a severely diseased sample and identification at strain-level resolution was approached as the number of sequencing reads was increased. We discuss how metagenomic sequencing could be implemented in routine plant disease diagnostics.

## Introduction

The sooner a disease outbreak is detected and the causative agent is identified, the faster the outbreak can be controlled by implementing testing, quarantine, and isolation. This applies to human, animal, and plant diseases^1^. Boxwood blight is a devastating fungal plant disease of ornamentals in the Buxaceae family including boxwood (*Buxus* spp.), sweet box (*Sarcococca* spp.), and pachysandra (*Pachysandra* spp.). Because boxwood is one of the most popular horticultural crops in the U.S. with annual sales of $126 million^2^, boxwood blight has caused significant economic losses and is of great concern to the landscape and nursery industry and home growers. The disease is caused by two closely related fungal species, *Calonectria pseudonaviculata* (*Cps*) and *Calonectria henricotiae* (*Che*). While *Cps* is widely distributed in North America, western Asia and Europe, *Che* has only been observed in Europe so far^3^. *Cps* was first detected in the U.S. in 2011 and has since been reported in at least 30 states^4^. Since *Cps* mainly spreads through infected plant material, contaminated tools, and other surfaces, early and rapid pathogen detection to avoid the distribution of infected plant material to home growers, nurseries, and public parks is critical to managing this disease.

Several diagnostic methods have been used for the detection of boxwood blight. Traditional morphology-based methods use observation of spores under the microscope. This requires expertise and a relatively long incubation period of the collected plant material because sporulation may need to be induced first^4^. In some cases, it is even necessary to isolate and culture the pathogen before spores can be observed. Moreover, spores of *Cps* and *Che* are so similar that their differentiation is challenging^5^ and there is even the risk that other fungi are mistaken for *Cps*^4^.

Molecular detection methods have been developed for faster and more sensitive detection of *Cps*. Polymerase chain reaction (PCR)-based assays are commonly used for direct detection of *Cps* and have been validated using environmental samples. However, in the early stages of assay development, these tests had a risk of false-positive signals^6^, and a trade-off between specificity and sensitivity in PCR-based assays has been found^7^. A set of new PCR-based protocols were developed to differentiate between *Cps* and *Che* but have only been validated on artificially inoculated plants^8^. Other molecular methods are based on Loop-mediated isothermal amplification (LAMP) and have been shown to exhibit high specificity for pure cultures. These assays can discriminate between the target pathogen and closely related species that may be present in the rhizosphere with no false-positive results. However, validation of *Cps* in rhizosphere samples gave negative results^9^. Finally, Next-generation sequencing (NGS) using Illumina technology has also been used to identify *Cps* as the pathogen causing Sarcococca blight. This method was able to identify *Calonectria* at the species-rank, but only after DNA was obtained from pure fungal cultures^10^.

Whole genome metagenomic sequencing is a promising new approach for pathogen detection and identification for disease diagnosis^11,12^. This culture-independent method consists in sequencing all DNA or RNA present in a sample, for example from a symptomatic host, and has been shown to provide accurate diagnosis. Since metagenomic sequencing does not rely on pathogen-specific probes or primers, little to no previous knowledge of the putative identity of the pathogen is required. In the case of boxwood, *Cps*, *Che*, and any other emerging bacterial, fungal, or oomycete boxwood pathogen could be identified. Metagenomics approaches utilizing NGS have been used in clinical research and are gradually being adopted in diagnosing plant diseases as well^13,14^. To achieve a rapid diagnosis, the MinION nanopore sequencer, a single‐molecule long-read sequencing platform developed by Oxford Nanopore Technologies Inc. (ONT) is particularly promising. It has several advantages over other NGS sequencing platforms: longer reads improve genome assembly and increase the precision of detection, first results are available minutes after a sequencing run is initiated, and it can be used almost anywhere, even in Space^15^. This portable sequencer has thus been used for metagenomic sequencing in medical research to successfully detect and sequence pathogens like Ebolavirus^16^ and SARS-CoV-2^17^.

However, the MinION has limitations regarding sensitivity and accuracy. Read accuracy is around 90%, which is lower than that of the short read technology Illumina. Although accuracy has recently been improved by increasing the accuracy with which raw signals obtained by the MinION are translated into base-pairs, a process called “base-calling”^18^. A more general challenge with metagenomics is that host genome sequences in the extracted DNA may represent the majority of the data^19^ and non-pathogenic microorganisms associated with the host plant may reduce the percentage of pathogen sequences further^20^, making it difficult to detect the causative agent.

With regard to plant disease diagnostics, metagenomic sequencing with the MinION using DNA or RNA extracted directly from plants enables rapid pathogen detection and identification in almost any laboratory or even in the field^20^. However, so far, the MinION has mainly been used to identify plant pathogenic viruses^21,22^ and bacteria^23,24^. Few studies have reported using the MinION for detection of plant pathogenic fungi^19,25^, which is challenging because of the poor representation of fungal genomes in reference databases and the technical difficulties in isolating high quality fungal DNA directly from plant tissue.

Here we applied metagenomic sequencing to the detection of *Cps* in naturally infected boxwood. The main objectives were to (i) find a DNA extraction method suitable for sequencing on the MinION and (ii) develop a bioinformatics workflow that optimizes detection sensitivity and specificity of the pathogen. While we focused on *Cps* and boxwood, the developed approach should be adaptable to most fungal pathogens of most plants and thus contribute to the improvement of plant disease diagnostics for outbreak control in general.

## Materials and methods

### Plant material

Naturally infected boxwood samples from various locations in Southwest Virginia were obtained from the Virginia Tech Plant Disease Clinic. Collection of plant material was done complying with institutional, national, and international guidelines and legislation. Samples were either moderately diseased or severely diseased (Supplementary Fig. 1). Healthy boxwood collected in the towns of Blacksburg and Floyd, Virginia, where no boxwood blight had been recorded at the time, served as negative controls. Plant material was stored at 4 °C for immediate use, otherwise at − 80 °C until DNA extraction.

### Extraction methods used to prepare DNA for MinION sequencing

**Figure 1:**
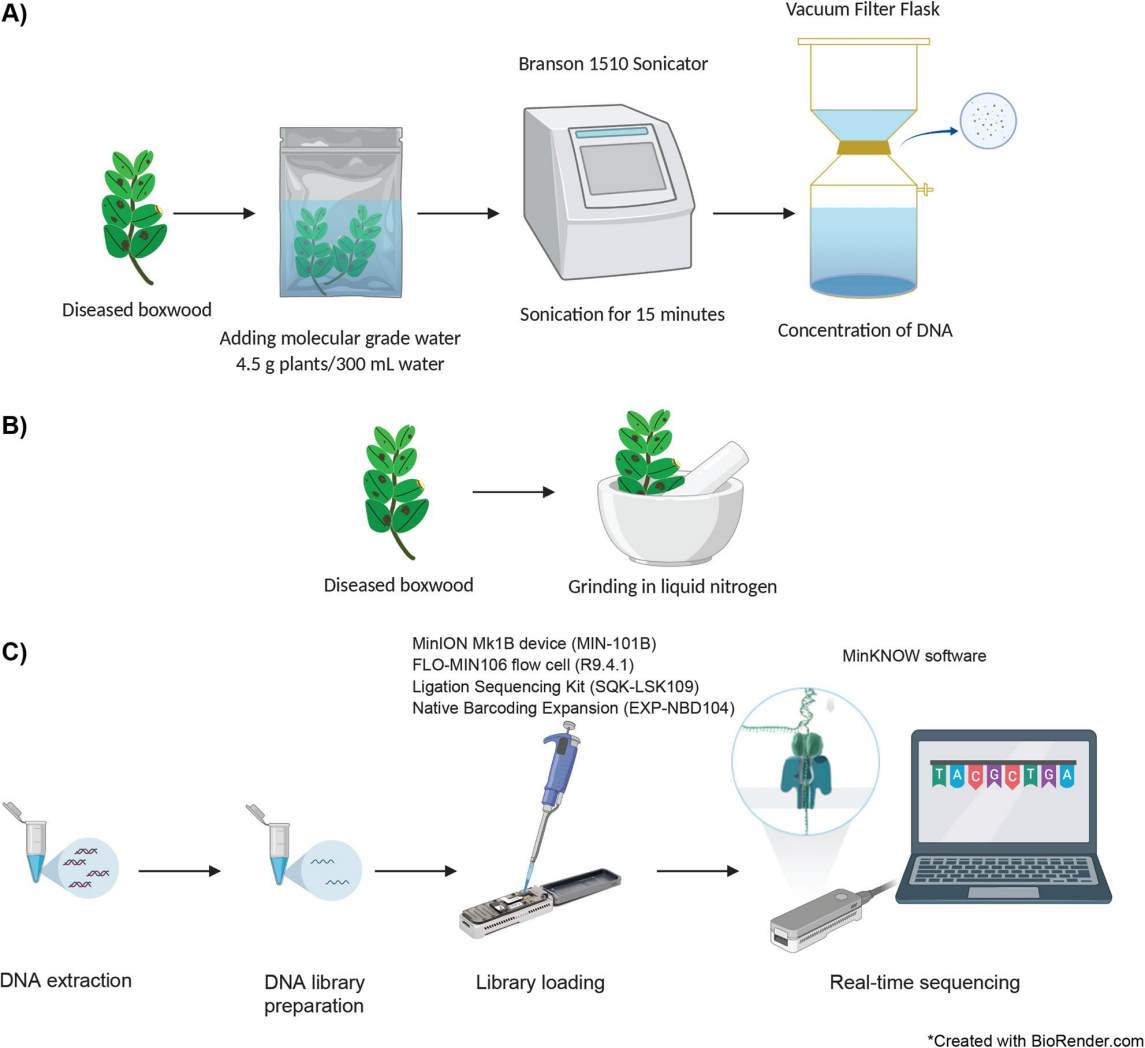
Pipelines for detection and identification of *Calonectria pseudonaviculata* (*Cps*). (**A**) DNA extraction approach based on sonication without disrupting plant cells. (**B**) DNA extraction approach based on homogenization in liquid nitrogen with disrupting plant cells. (**C**) The MinION sequencing pipeline. Created with BioRender.com.

To determine the most efficient DNA extraction method, both moderately and severely diseased samples were either sonicated (without disrupting plant cells) or homogenized in liquid nitrogen (disrupting plant cells) (Fig. 1). DNA was measured using a Thermo Scientific NanoDrop spectrophotometer.

For sonication, 4.5 g of plant tissue composed of twigs of moderately diseased or severely diseased plants were placed in a Ziploc bag containing nuclease-free water. Next, the bag was sonicated for 15 min to dislodge as many microorganisms as possible from the plant into the liquid and disrupt their cells. The liquid went through a vacuum filter flask to concentrate DNA on the filter membrane. DNA was extracted from the membrane using kits designed for water and soil samples, as shown in Table 1 (sample IDs starting with the letter S).

**Table 1:** Metadata and DNA quantity and quality of samples used in this study.

Sample description	Sample ID	Sequencing date	Extraction methods	Kit	Flowcell ID	Number of barcodes used per flowcell	DNA quantity and quality		
							DNA concentration (ng/μL)	A260/A280	A260/A230
Moderately diseased plant	S1	Nov22	Sonication	DNeasy^®^ PowerWater^®^	FAK95928	4	317.0	1.90	1.98
	S2	Nov22	Sonication	DNeasy^®^ PowerSoil^®^ Pro			479.5	1.93	1.74
	S3	Nov22	Sonication	ZymoBIOMICS™ DNA Miniprep			403.5	1.92	1.96
	G1	Nov22	Grinding	ZymoBIOMICS™ DNA Miniprep			76.0	1.89	1.72
	G2	Nov24	Grinding	DNeasy^®^ PowerPlant^®^ Pro	FAK96453	5	163.1	1.66	0.74
	G3	Nov24	Grinding	Invisorb^®^ Spin Plant Mini			103.0	1.71	0.57
	G4	Nov24	Grinding	OmniPrep™			203.1	1.73	0.69
	G5	Nov24	Grinding	OmniPrep™ with RNAse			277.8	1.81	1.95
	G6	Nov24	Grinding	Gentra^®^ Puregene^®^			314.6	1.45	0.45
Severely diseased plant (Batch 1)	S4	Dec12	Sonication	DNeasy^®^ PowerWater^®^	FAN08223	4	151.0	1.89	2.02
	S5	Dec12	Sonication	DNeasy^®^ PowerSoil^®^ Pro			98.0	1.90	0.83
	G7	Dec12	Grinding	DNeasy^®^ PowerPlant^®^ Pro			76.8	0.89	0.20
	G8	Dec12	Grinding	Invisorb^®^ Spin Plant Mini			135.1	1.77	1.49
	G9	Dec17	Grinding	OmniPrep™	FAN08200	5	170.3	2.10	2.23
Severely diseased plant (Batch 2)	G10	Dec17	Grinding	OmniPrep™			1132.5	2.10	1.88
	G11	Dec17	Grinding	OmniPrep™ diluted 10 times and treated with Rnase			110.1	1.96	0.49
	G12	Dec17	Grinding	ZymoBIOMICS™ DNA Miniprep			77.4	2.19	0.08
	G13	Dec17	Grinding	Gentra^®^ Puregene^®^			349.0	1.57	0.55
Healthy plant	NC	Negative control	Grinding	ZymoBIOMICS™ DNA Miniprep	FAO99127	2	82.9	1.87	1.53

For homogenization, plant tissue composed of leaves and stems randomly picked from moderately diseased or severely diseased plants was ground in liquid nitrogen. 0.1 g of ground tissue was used for DNA extraction using kits as shown in Table 1 (sample IDs starting with the letter G). For extraction from severely diseased plant batch 1, 0.1 g of severely diseased boxwood was ground and processed individually for each DNA extraction. However, to make plant samples more similar to each other and results obtained with different kits more comparable, this was changed for the later batches: several grams of tissue were ground together and then 0.1 g aliquots were used for individual DNA extractions. For the negative control, DNA was extracted with the ZymoBIOMICS DNA Miniprep Kit from a 0.1 g aliquot of ground, healthy plant tissue (sample ID: NC).

### MinION library preparation and sequencing

MinION Library preparation was performed according to the native barcoding genomic DNA protocol (EXP-NBD104, EXPNBD114, and SQK-LSK109)^26^ with minor modifications. The library was prepared using the Ligation Sequencing Kit (ONT; SQK-LSK109). For each run, first, DNA for each sample was repaired and end-prepped for each sample using the NEBNext Ultra II End Repair/dA-Tailing Module (New England Biolabs, Inc.; Catalog # E7546S). 90 μL AMPure XP beads were used for cleaning up repaired DNA. Then repaired DNA was washed on a magnetic rack using freshly made 70% ethanol and eluted with 25 μL nuclease-free water. Second, native barcode ligation was performed by mixing 22.5 μL of the elute with the Blunt/TA Ligase Master Mix (New England Biolabs, Inc.; Catalog # M0367S) and Native Barcode (ONT; Native Barcoding Expansion Kit EXP-NBD104). Barcoded DNA was cleaned up by another wash step using 90 μL AMPure XP beads, and DNA was eluted in 26 μL nuclease-free water. Then equimolar amounts of each barcoded DNA were pooled into a 1.5 mL microcentrifuge tube. Last, adapter ligation was performed by mixing the pooled barcoded sample with Adapter Mix (ONT; SQK-LSK109), NEBNext Quick Ligation Reaction Buffer (New England Biolabs, Inc.; Catalog # B6058S) and Quick T4 DNA Ligase (New England Biolabs, Inc.; Catalog # M2200S). Ligated DNA was cleaned up with 60 μL AMPure XP beads, washed on a magnetic rack using Long Fragment Buffer (ONT; SQK-LSK109), and eluted with 15 μL Elution Buffer (ONT; SQK-LSK109).

Sequencing reactions were performed independently for each run on a MinION flow cell (ONT; FLO-MIN106 R9.4.1 Version) connected to a Mk1B device (ONT; MIN-101B) operated by the MinKNOW software (ONT, Inc. v19.12.2). Each flow cell was primed with the priming buffer prepared by mixing 30 μL Flush Tether (ONT; EXP-FLP002) with a tube of Flush Buffer (ONT; EXP-FLP002). 12 μL of the final library mixed with Sequencing Buffer (ONT; SQK-LSK109) and Library Loading Beads (ONT; SQK-LSK109) were loaded onto the SpotON sample port of the flow cell in a dropwise fashion. The sequencing run was stopped when all pores lost activity, usually after 48–72 h. A new flow cell was used for each run. Sample IDs and descriptions are shown in Table 1. After sequencing, the raw files in FAST5 format, containing the electrical signals, were translated (base-called) with the ONT tool Guppy GPU (v3.2.2) into sequences with a minimum q-score of 7 and saved as FASTQ files for further analysis. The FASTQ files were then converted to FASTA files with an in-house shell script.

### DNA extraction and Illumina sequencing

Healthy plant tissue (100 mg) and severely diseased plant tissue (100 mg) were homogenized in liquid nitrogen for DNA extraction for Illumina sequencing to serve as controls for MinION sequencing. DNA of healthy boxwood was extracted using Invisorb Spin Plant Mini Kit, and DNA of severely diseased boxwood was extracted using ZymoBIOMICS DNA Miniprep Kit.

Whole-genome sequencing of healthy boxwood was performed on an Illumina Nova Seq 6000 Platform (2 × 150 bp) at Novogene Corporation Inc. (Sacramento, CA). Low-quality reads and adapters were removed by the company. Illumina sequencing of severely diseased plant tissue was performed on an Illumina HiSeq 3000 Platform (2 × 100 bp) at the Iowa State University DNA Facility using six out of 96 barcodes (thus using 6/96th of a single run), and the quality of reads was checked using FastQC v0.11.9^27^. Reads were trimmed using Trimmomatic v0.39^28^ to remove adapters.

### Metagenomic analysis

Two custom fungal genome databases were constructed for taxonomic assignment of fungal reads. First, to determine the DNA extraction method that yields the highest percentage of *Cps*, a small database containing only four fungal genomes of the family Nectriaceae was constructed: *Cps* CBS 139395, *Che* CBS 138102, *Fusarium graminearum* PH-1, and *Pseudonectria foliicola* AR2711 (downloaded from NCBI). The *Cps* genome was used to identify *Cps* reads and the Volutella blight pathogen *Pseudonectria foliicola* was included since it frequently co-infects boxwood with *Cps*. The *Che* genome was added as the negative control since it is closely related to *Cps* but is not present in the USA and the *F. graminearum* genome served as the second negative control since it is another member of the family Nectriaceae but does not cause disease on boxwood. A more extensive database (referred to as large database from here on) was used for a more in-depth characterization of the obtained metagenomes: all assembled genomes of *Cps*, *Che*, *F. graminearum*, *P foliicola* and *Pseudonectria buxi* (another Volutella blight pathogen) available at NCBI in April 2021 (Supplementary Table 1). Reads were trimmed with Porechop v0.2.4^29^ to remove adapters before using them with this database.

Three bioinformatics tools for taxonomic assignment of MinION readswere used: 1. BLASTN v2.10.0 +^30^, 2. MetaMaps v0.1^31^, and 3. Kraken 2 v2.1.1^32^. BLASTN was chosen because it is a commonly used tool to identify fungi^33^. The E-value parameter was set to less than 0.001, and results were filtered for alignments longer than 1000 bp. For each read, the hit with the lowest E-value was used for taxonomic assignment. MetaMaps was specifically developed for taxonomic assignment of long metagenomic reads^31^. The parameter --perc_identity was set to 85, and hits were further filtered to an identity greater than 85% since hits with lower percentage identity were still reported even using the --perc_identity 85 parameter. Since MetaMaps provides a single taxonomic assignment for each read, ranking was not necessary. Kraken 2 is a popular tool for taxonomic read assignment that provides high accuracy and has faster speeds and lower memory requirements than the original Kraken^32,34^. It has been shown to work well for MinION reads^35^ but was originally designed for short reads and was thus used for both MinION and Illumina reads. The default parameters were used for MinION reads, and the parameter --paired was used for Illumina reads.

Since contigs derived from assembled reads have a lower error rate than raw reads, *Cps* genomes were assembled to attempt identification of the *Cps* lineage present in our sample. *Cps* reads that had been pre-identified by BLASTN in samples G10, G11 and G12 using the extensive database were used as input. Canu v2.1.1^36^ was used for assembly and QUAST v5.0.2^37^ and BUSCO v5.0.0^38^ were used to assess the quality of the assembled *Cps* genome. CBS139395 served as the reference genome for QUAST. BUSCO was based on the lineage-specific profile library hypocreales_odb10. To explore strain-level identification, BLASTN and sourmash v4.0.0^39^ were then used in parallel to determine the similarity between the genome assemblies and the reference *Cps* genomes. For sourmash, the parameters -p, scaled = 1000, and k = 21 were used for generating signatures of the assembly and the reference genomes with the sketch dna command. The search command was then used to identify which *Cps* genome in the database was most similar to the assemblies (measured as Jaccard similarity). For BLASTN, the same parameters as in the previous sections were used.

To determine the minimal number of MinION reads required to consistently detect *Cps* in a subset of the obtained samples, reads were randomly sub-sampled 10 times at each of the following sub-sample sizes: 200, 300, 500, 700, and 1000. For each sub-sample, BLASTN hits for *Cps* were retrieved using the read IDs and counted.

All programs were run on Virginia Tech’s high performance computer network ARC. For data visualization, R was used to generate the bubble plot. KronaTools v2.7.1^40^ was used to generate graphical interactive html taxonomy abundance piecharts.

## Results

### Experimental design overview

To determine the feasibility of culture-independent metagenomics for detection of the boxwood pathogen *Cps*, several DNA extraction methods, two DNA sequencing technologies, and several bioinformatics metagenomics analysis tools were used in parallel. Because it was not feasible to test all combinations of protocols and tools, experiments and respective results were grouped as follows: (1) Identification of DNA extraction methods that provide DNA of sufficient quantity and quality for ONT MinION sequencing and a high percentage of *Cps* sequencing reads based on the analysis of all samples sequenced with the ONT MinION using two metagenomics tools and a small fungal reference database; (2) *Cps* identification using additional bioinformatics tools in combination with a large fungal genome database; (3) Comparison of results obtained with the ONT MinION to results obtained with the Illumina sequencing platforms using a bioinformatics tool that can be used for both platforms; (4) Attempt at lineage-specific *Cps* identification after assembling sequencing reads; (5) Determination of the smallest number of MinION reads necessary to detect *Cps* in severely diseased samples.

### DNA extraction from either ground boxwood tissue or wash water of sonicated tissue is adequate for detection of *Cps*

Two fundamentally different DNA extraction methods were tested: extraction of DNA from wash water of relatively large sonicated plant samples (4.5 g) and DNA extraction from a relatively small amount of plant tissue (0.1 g) that was ground in liquid nitrogen (Fig. 1). The rationale was that sonication can be expected to maximize the DNA of microorganisms that are easily separated from the host plant and should thus minimize contaminating plant DNA, whereas homogenization in liquid nitrogen efficiently frees DNA from all cells (plant, prokaryotic, and fungal) and can thus be expected to increase fungal DNA yield while also increasing plant DNA contamination.

Both extraction methods and all kits resulted in more than 1 µg per sample, which is the required minimum for use with the ONT MinION native barcoding genomic DNA protocol. DNA concentrations ranged widely from 76 ng/µL to over 1133 ng/µL, but the majority of DNA extractions using either grinding or sonication yielded DNA concentrations in the range from 100 to 500 ng/µl and were similarly effective for both moderately and severely diseased samples (Table 1).

With regard to quality, we determined the A260/A280 (DNA/protein) and A260/A230 (DNA/other impurities) ratios, which for pure DNA are expected to be around 1.8 and 2.0–2.2, respectively. A260/A280 ratios were close to 1.8 for most samples independent of extraction method and severity of disease (with the exception of one DNA sample extracted from a ground severely diseased sample, which had a ratio of only 0.89), suggesting low protein contamination in most samples. The A260/A230 ratio instead varied widely from almost 0 to 2.2, and DNA extracted from ground samples had generally lower ratios than DNA extracted from wash water after sonication, suggesting that more impurities were present in DNA extracted from ground samples. Severity of disease did not appear to affect the A260/A230 ratio.

Next, we analyzed the overall DNA sequencing output focusing on the total length of reads and the number of reads obtained per sample (Table 2). Since a different number of barcoded samples was sequenced on different flow cells, we also computed the total read length and number of reads that we would have obtained if we had used an entire flow cell for each sample. Normalized read length/flow cell varied between 5.4 to 26.2 gigabases (Gb) for DNA extracted from wash water of sonicated samples and between 2.9 and 22.9 Gb for DNA extracted from ground samples. The normalized number of reads/flow cell varied similarly widely between 1.4 to 11.4 million (M) for DNA extracted from wash water of sonicated samples and between 1.4 and 19.2 M for DNA extracted from ground samples. Also, average read length and the length of the longest read varied widely for both extraction methods. As with DNA concentration and quality, severity of disease did not affect overall sequencing results. In summary, all extraction methods and kits were comparable in regard to overall DNA sequencing metrics and, unexpectedly, sequencing results did not correlate with either DNA concentration or DNA quality.

**Table 2:** Summary of ONT MinION sequencing data obtained in this study (see Table 1 for sample metadata).

ID	Total read length (Gbp)	Normalized read length per flow cell (Gbp)	Total number of reads	Normalized number of reads per flow cell	Average read length (bp)	Longest read length (bp)	Number of *Cps* hits ≥ 1000 bp (based on BLASTN)	Number of *Cps* hits ≥ 85% id (based on MetaMaps)	Total read length of *Cps* hits ≥ 1000 bp (Mbp; based on BLASTN)	*Cps* reads (based on BLASTN) out of total reads (%)	*Cps* (based on BLASTN) read length out of total read length (%)	*Cps* genome coverage (×)
S1	1.35	5.41	429,098	1,716,392	3152	89,153	174	86	0.64	0.04	0.05	0.012
S2	1.92	7.68	475,383	1,901,532	4040	91,256	166	87	0.75	0.03	0.04	0.014
S3	1.37	5.49	354,893	1,419,572	3864	65,510	349	192	1.16	0.10	0.08	0.021
G1	1.30	5.21	711,491	2,845,964	1830	54,580	6382	3548	17.33	0.90	1.33	0.315
G2	2.55	12.73	2,020,441	10,102,205	1260	54,580	18,797	9269	46.68	0.93	1.83	0.849
G3	1.97	9.84	1,965,416	9,827,080	1001	88,418	8528	4056	19.82	0.43	1.01	0.360
G4	2.91	14.57	2,724,170	13,620,850	1069	64,110	4859	1841	9.67	0.18	0.33	0.176
G5	4.56	22.88	3,843,496	19,217,480	1190	72,917	9977	4271	20.98	0.26	0.46	0.381
G6	0.60	2.98	468,312	2,341,560	1274	50,579	3027	1190	6.82	0.65	1.14	0.124
S4	4.90	19.59	2,430,505	9,722,020	2014	64,015	3681	1700	9.52	0.15	0.19	0.173
S5	6.55	26.19	2,846,336	11,385,344	2300	189,652	9379	3763	22.78	0.13	0.35	0.414
G7	1.14	4.58	1,399,778	5,599,112	817	42,603	11,987	8255	36.71	0.86	3.21	0.668
G8	2.31	9.24	2,839,930	11,359,720	813	369,167	13,146	7697	38.09	0.46	1.65	0.693
G9	1.14	5.69	298,982	1,494,910	3804	77,811	14,484	10,343	46.02	4.84	4.05	0.837
G10	2.06	10.30	549,134	2,745,670	3749	73,800	46,460	40,409	257.82	8.46	12.52	4.670
G11	0.92	4.61	292,084	1,460,420	3154	40,849	27,566	22,881	110.97	9.44	12.04	2.019
G12	3.46	17.31	894,828	4,474,140	3868	53,435	65,192	59,892	386.05	7.29	11.15	7.022
G13	0.74	3.70	280,861	1,404,305	2633	91,251	11,526	9677	67.72	4.10	9.15	1.232
NC	2.10	4.19	846,387	1,692,774	2476	28,907	0	0	0	0.00	0.00	0

*Gbp* giga base pairs, *Mbp* mega base pairs.

**Figure 2:**
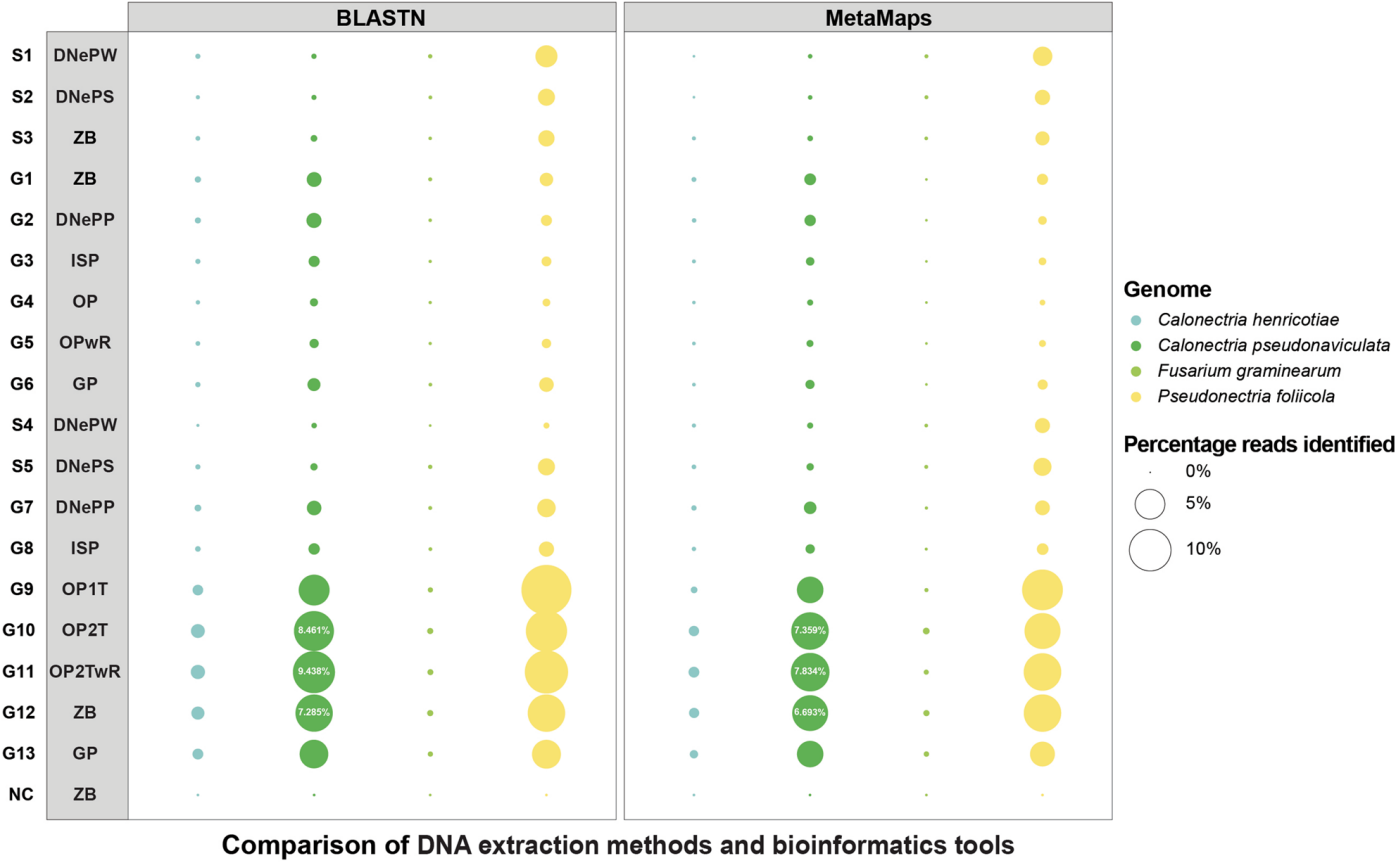
Bubble plot showing the percentage of sequencing reads assigned to four fungal species in each sequenced sample. The column on the left displays the sample IDs and the column to its right displays the abbreviations of DNA extraction kits (see Table 1). Bubble size is proportional to the percentage of reads assigned to the four species listed on the right based on the tools BLASTN and MetaMaps using a small fungal database containing one genome per fungal species.

Finally, sequencing results were analyzed for the presence of *Cps* sequences. To do this, reads were classified taxonomically using two independent tools in parallel, BLASTN and MetaMaps, and a small fungal reference library containing one *Cps* genome and one genome each of three additional species in the Nectriaceae family. While BLASTN generally identified twice as many reads as *Cps* compared to MetaMaps (Table 2 and Fig. 2), the relative number of *Cps* reads between individual samples was the same for both tools, giving confidence that either tool could be used to compare samples with each other. Since BLASTN is the more widely used tool out of the two, only BLASTN results are reported in the next paragraphs.

Since samples differed from each other in the number of reads and total read length, we determined (1) the percentage of reads assigned to *Cps* out of all reads per sample (Table 2 and Fig. 2) and (2) the percentage of the total length of reads identified as *Cps* out of the total length of reads per sample (Table 2). With regard to read number, DNA extracted from ground samples recovered a higher percentage of *Cps* reads (up to 9.44%) compared to DNA extracted from sonicated samples (only up to 0.15%). With regard to the percentage of the total length of *Cps* sequences out of the total sequencing length, DNA extracted from ground samples gave percentages of up to 12.52% while sonicated samples only gave percentages up to 0.35%. However, two samples obtained from ground tissue (G7 and G8) of the severely diseased batch 1 also had low percentages of *Cps* with regard to read number and length.

We cannot make any conclusions on individual DNA purification kits because most kits were only used once with moderately diseased boxwood samples and once with severely diseased boxwood samples. Additionally, DNA was sequenced on four separate flow cells (which quality is known to be inconsistent, in particular, with regard to the number of active pores). Importantly though, all kits performed sufficiently well to allow for downstream *Cps* detection.

As expected, a higher percentage of *Cps* reads was obtained from severely diseased samples (up to 9.44%) than from moderately diseased samples (up to 0.93%). Importantly, not a single *Cps* read was found in the negative control DNA extracted from a healthy boxwood plant. With regard to the other fungal species included in the reference library, only a very small number of reads of *Che* and *Fusarium graminearum* were recovered. When the reads identified as *Che* using our small reference library were compared by BLASTN against the entire nt database at NCBI^41^, these reads were more similar to other fungi or bacteria than to *Che* and were thus false positives. The ubiquitous boxwood pathogen *Pseudonectria foliicola* was found in all diseased samples in percentages similar or even higher than *Cps* but not in the healthy boxwood sample.

### Robust *Cps* identification using BLASTN and Kraken 2 in combination with an expanded Nectriaceae genome database

For a more in-depth characterization of *Cps* and the other Nectriaceae family members in the metagenomic sequences, a large database containing all public genome assemblies of *Cps*, *Che*, *P. foliicola*, *P. buxi*, and *F. graminearum* was used. Although we had used BLASTN and MetaMaps to identify the best DNA extraction methods above, we replaced MetaMaps with Kraken 2^32^ here. Compared to MetaMaps, Kraken 2 has been used more widely in published metagenomic studies, is user-friendly, and has been shown to have high accuracy, low memory usage, and high speeds^32,34^.

First, species-level taxonomic classification results obtained with Kraken 2 were compared with those obtained with BLASTN and showed that Kraken 2 also identified *Cps* in all diseased samples (Supplementary Table 2). Kraken 2 classified an even higher number of reads as one of the five fungal species present in the reference database than BLASTN. For example, Kraken 2 classified 26.62% of total reads in G10 as belonging to the five fungal species while BLASTN only 20.75%. For the moderately diseased samples from which DNA was extracted after sonication, Kraken 2 identified 0.05 to ~ 0.11% of total reads as *Cps* (Supplementary Fig. 2).

**Figure 3:**
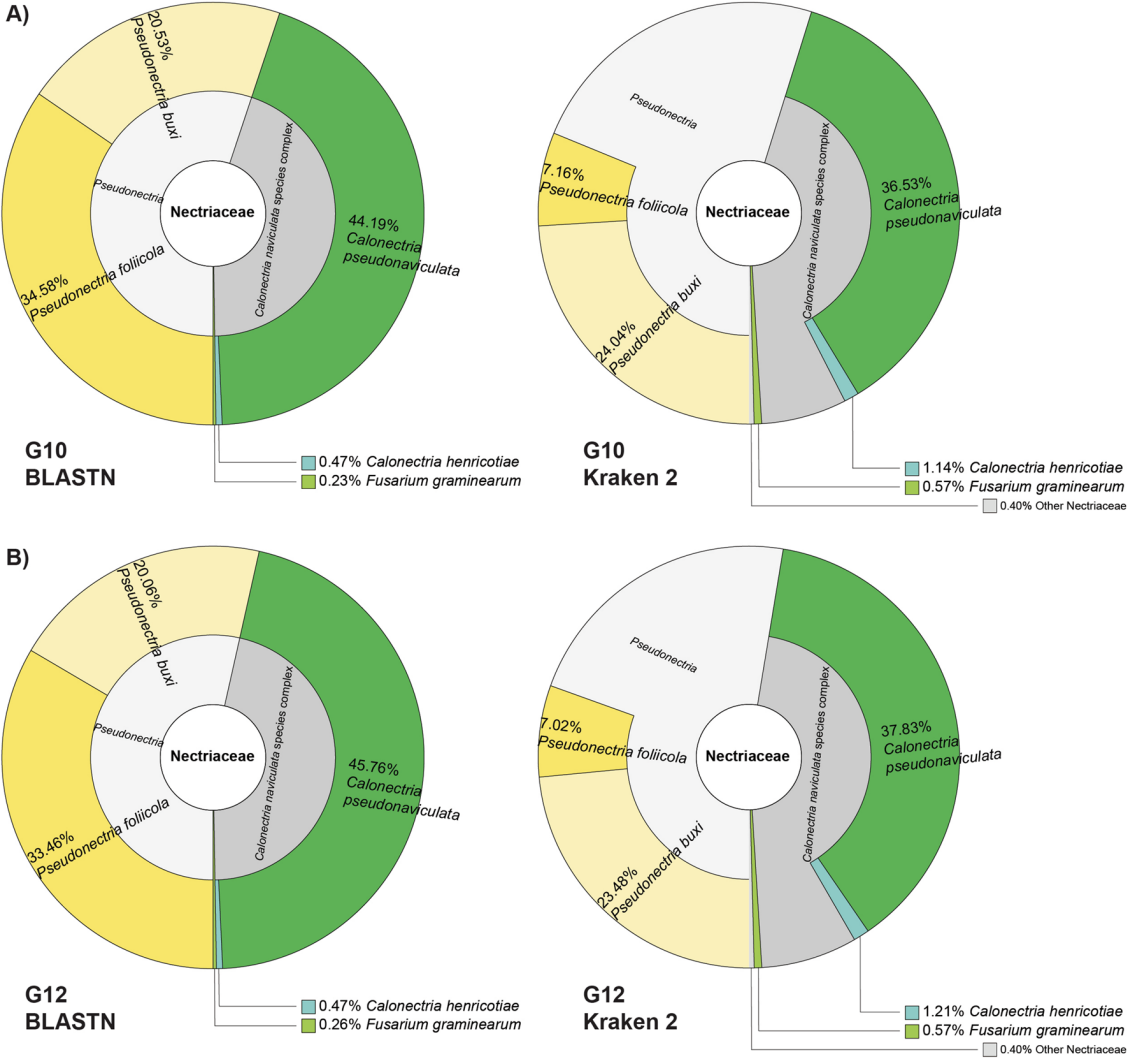
Krona plots showing the fraction of reads identified at the species, species complex, or genus rank as a percentage of the sequencing reads assigned to the family Nectriaceae using the tool Kraken 2 and a database of 29 genomes. The plots on the left display BLASTN results and the ones on the right Kraken 2 results. Each color represents a species, species complex, or genus. (**A**) Results of G10, the sample processed by OmniPrep after homogenization in liquid nitrogen. (**B**) Results of G12, the sample processed by ZymoBIOMICS DNA Miniprep Kit after homogenization in liquid nitrogen (see Table 1).

When looking specifically at *Cps*, 36.53% of all reads assigned to one of the five Nectriaceae species in sample G10 were identified as *Cps* by Kraken 2, whereas 44.19% were identified as *Cps* by BLASTN (Fig. 3). For G12, 37.83% of fungal reads were identified as *Cps* by Kraken 2, whereas 45.76% were identified as *Cps* by BLASTN. This difference is due to the fact that Kraken 2 classified a subset of *Calonectria* reads at the *Calonectria* species complex rank without assigning them to an individual species, but our BLASTN pipeline assigned all fungal reads at the species rank.

Also when using the large reference database, a small subset of reads was identified as *Che* by both BLASTN and Kraken 2. However, these reads matched bacterial, yeast, or plant sequences when compared against NCBI’s nt database (Supplementary Table 3 shows the results for sample S1 as example). The most remarkable new result using the large fungal database was the identification of the Volutella pathogen species *P. buxi* at an abundance similar to *P. foliicola*. The *P. buxi* reads were probably identified as *P. foliicola* when using the small database since *P. buxi* was not included in the smaller database. As with *Calonectria*, Kraken 2 classified some reads as *Pseudovaniculata* without species designation, while our BLASTN pipeline assigned all *Pseudovaniculata* reads to either *P. foliicola* or *P. buxi*. Approximately 0.5% of fungal reads in G10 and G12 were identified as *F. graminearum* but may belong to related *Fusarium* species since only *F. graminearum* genomes were included in the database, and it was thus not possible to distinguish between individual *Fusarium* species.

Unexpectedly, a small number of reads were identified as *Cps* by both Kraken 2 and BLASTN in the healthy negative control sample. Still, as the *Che* reads above, they were identified as false positives when comparing them to NCBI’s nt database^41^.

### MinION and Illumina sequencing provide similar results in regard to *Cps* identification

**Figure 4:**
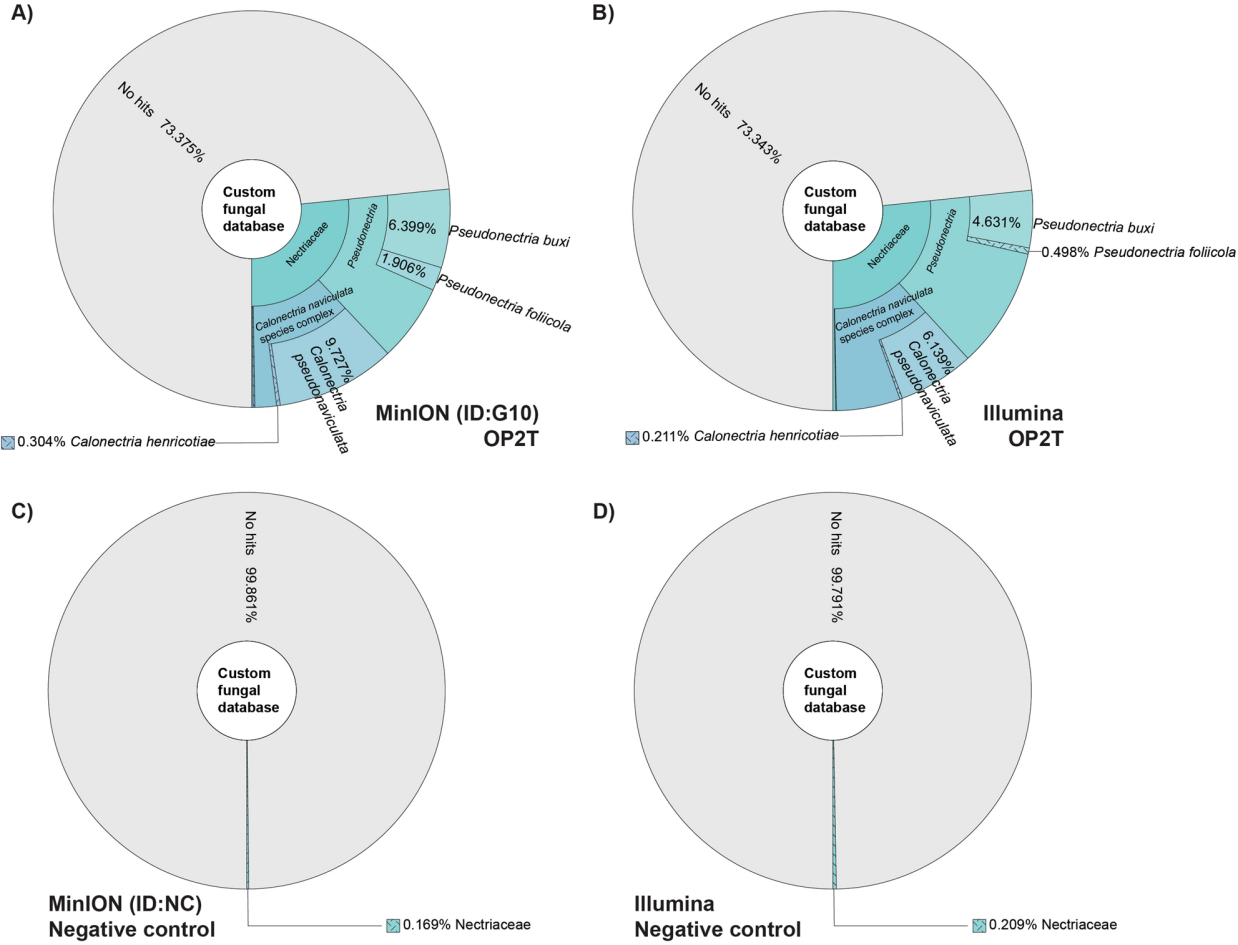
Krona plots showing the fraction of reads identified as members of the family Nectriaceae as a percentage of all sequencing reads using the tool Kraken 2 and a database of 29 genomes. Each color represents a clade. (**A**) Results of G10 sequenced on the ONT MinION. (**B**) Results of G10 sequenced on the Illumina HiSeq 3000 platform. (**C**) Results of a healthy sample sequenced on the ONT MinION. (**D**) Results of another healthy sample sequenced on the Illumina Nova Seq 6000 Platform.

To compare the results of ONT MinION long-read sequencing with the Illumina short-read platform, sample G10 and a negative control sample were sequenced using Illumina technology. Since Kraken 2 can be used for both short- and long-reads^35,42^, we used Kraken 2 in combination with our large fungal database to compare the results from the two sequencing platforms. Illumina sequencing yielded 17,033,700 paired-end reads with a total length of 1.50 Gb compared to the 541,576 long reads with a total length of 1.96 Gb obtained by MinION sequencing (Supplementary Table 4). 9.73% of MinION reads and 6.14% of Illumina reads were identified as *Cps*, respectively (Fig. 4). The lower percentage of Illumina reads identified as *Cps* was compensated by the higher percentage of Illumina reads that were assigned to the *Calonectria naviculata* species complex without species identification.

Since we had no DNA of the healthy boxwood left that we had used as the negative control for MinION sequencing, a different DNA sample of a healthy boxwood was sequenced with Illumina. Illumina sequencing yielded 271,857,762 paired-end reads with a total length of 40,778,664,300 bp per sample (Supplementary Table 4). As for the healthy negative control sample used with MinION sequencing, a very small number of reads of this sample were assigned to *Cps* (Fig. 4). However, when these reads were compared with the entire nt database at NCBI using BLASTN, they were again found to be false positives.

### *Cps* in diseased plants can be identified to a within-species cluster using sourmash and BLASTN

In a recent study, investigating the emergence of boxwood blight using population genomics, several clusters/lineages within the *Cps* species were identified^43^. Therefore, we wanted to determine if *Cps* reads in our samples could be assigned to one of the identified clusters. Since the program sourmash can identify bacterial genomes in metagenomes independently of taxonomy and without the need for NCBI taxonomic identifiers, we first attempted to use sourmash using all reads of samples G10, G11, and G12 as query and the same extended fungal database we had used with Kraken 2, but sourmash did not identify any fungal genome in any of the samples. However, Table 3 shows that when using only the reads that had been identified as *Cps* by BLASTN as query, sourmash did find them to have similarity to *Cps* genomes. The highest similarity was to the genomes of *Cps* isolates CBS139394 and CBS139395 (both isolated from sweet box in Maryland, USA^10^) followed by genome sequences of isolate CB002 (isolated from boxwood in Belgium^5^). Similarity was unexpectedly low (14–19%). Since the low similarity could have been due to sequencing errors present in individual reads, we then assembled all *Cps* reads from G10, G11, and G12 with the expectation that the assembled reads would have fewer errors and be more similar to the reference genomes. The *Cps* genome we obtained was 49,048,547 bp long and consisted of 1055 contigs. 48,291,239 bp of the assembly aligned with 88.746% of the chosen reference genome CBS139395 (Table 4). Although this revealed that our assembly covered most of the *Cps* genome, only 50.3% of genes were complete and 23.2% were fragmented compared to 96.6% of genes that were complete in the reference genome CBS139395 based on BUSCO^38^ assessment (Table 4). When the assembled genome was used as query with sourmash against our fungal database, the genomes CBS139394, CBS139395, and CBS002 were again found to be most similar, but now with a similarity value close to 73% (Table 3). When using BLASTN, the assembled *Cps* genome had a significantly higher number of best hits to CBS139395 than to all other genomes (Table 3).

**Table 3:** Percentage of *Cps* based on Jaccard similarity obtained with sourmash and *Cps* hits obtained with BLASTN.

Reference genome	Accession number	G10 *Cps* reads	G11 *Cps* reads	G12 *Cps* reads	Assembled *Cps* genome	
		Similarity (%) by sourmash	Similarity (%) by sourmash	Similarity (%) by sourmash	Similarity (%) by sourmash	Number of hits by BLASTN
CBS139395	GCA_004380915.1	17.47	19.11	14.21%	72.27%	621
CBS139394	GCA_001696505.1	17.30	18.93	14.01%	72.65%	125
CB002	GCA_006505905.1	17.11	18.72	13.83%	72.53%	3
	GCA_004141935.1	17.11	18.72	13.83%	72.53%	
CT13	GCA_004380985.1	16.65	18.26%	13.36%	72.12%	217
CBS14417	GCA_004381005.1	16.51	18.10%	13.23%	72.02%	43
ODA1	GCA_004382225.1	15.69	17.36%	12.53%	68.81%	31
NC-BB1	GCA_004381035.1	13.85	15.54%	11.02%	61.20%	5
ICMP14368	GCA_004382245.1	10.73	12.56%	8.48%	48.09%	5

**Table 4:** Assembly summary of assembled *Cps* reads that were pre-identified by BLASTN in samples G10, G11 and G12, and of reference genome CBS139395.

	Assembled *Cps*	CBS139395
Assembly size (bp)	49,048,547	54,975,240
Number of contigs	1,055	27
Maximum contig length (bp)	419,837	5,578,780
N50 contig length (bp)	88,131	3,534,399
GC content (%)	48.12	46.36
Total aligned length (bp)	48,291,239	NA
Genome fraction (%)	88.746	NA
^a^Assembly BUSCO coverage (%)	C:50.3; F:23.2; M:26.5	C:96.6; F:0.2; M:3.2

^a^For BUSCO coverage, C stands for complete BUSCOs, F stands for fragmented BUSCOs, and M stands for missing BUSCOs.

### *Cps* was detected in as few as 200 sub-sampled MinION sequencing reads in severely diseased tissue

**Figure 5:**
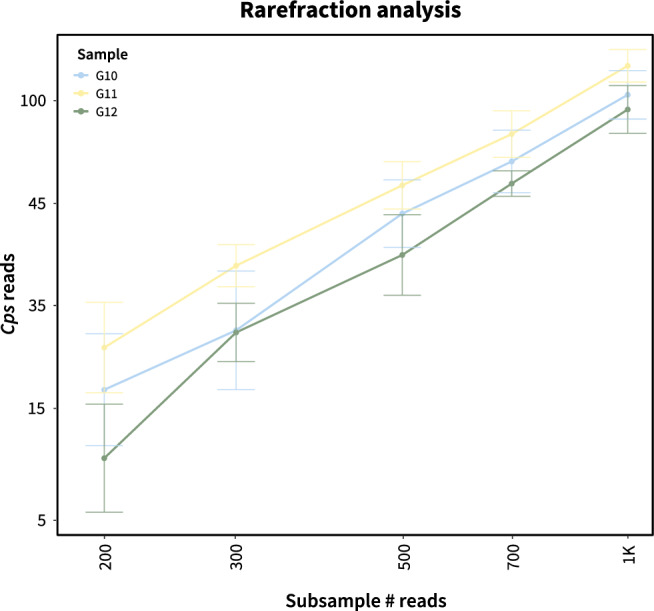
Detection limit analysis based on computational sub-sampling. Sub-samples were obtained by randomly extracting reads from original sequencing files. The X-axis shows the number of sub-sampled reads. The Y-axis shows the number of identified *Cps* reads. The circles represent the median value for each sub-sample size and error bars show the standard deviation among the 10 subsampling events.

After showing that *Cps* can be identified with high specificity from naturally infected boxwood tissue using metagenomic sequencing with the ONT MinION, we wanted to investigate the minimal number of reads needed to detect *Cps*. We thus computationally sub-sampled samples G10, G11, and G12 to different read numbers generating 10 random subsamples for each size shown in Fig. 5. Importantly, even for the sub-samples consisting of only 200 total reads, there was not a single sub-sample in either G10, G11, or G12 without *Cps* reads (Fig. 5).

## Discussion

Sensitive, specific, and fast pathogen detection is instrumental in plant disease control and management. Here we explored metagenomic sequencing using the ONT MinION and Illumina for detection and identification of the boxwood blight pathogen *Cps*.

To effectively use metagenomics for *Cps* detection, we first needed to identify a suitable DNA extraction method. We tested two protocols. One protocol aimed at minimizing host DNA by not disrupting host cells and assuming *Cps* could be separated from host tissue by washing and sonication. The other protocol was designed to obtain as much total DNA as possible by disrupting both host cells and fungal cells by grinding in liquid nitrogen. For most samples, disrupting host cells yielded more *Cps* sequencing reads than not disrupting host cells. This indicates that most *Cps* is likely to be embedded in host tissues upon infection, while only a small amount of *Cps* exists on the host surface. However, for all samples, *Cps* reads were identified even in DNA extracted from wash water of sonicated tissue revealing that both protocols can be used to prepare DNA for metagenomic sequencing.

Compared to results using metagenomic sequencing for the identification of bacterial plant pathogens, the recovery of fungal pathogen reads in this study was relatively low. In fact, up to 60% of reads were identified as the bacterial pathogen *Xanthomonas perforans* in tomato plants naturally infected with bacterial spot^23^. However, for fungal plant pathogens, other studies reported recovery of very few pathogen reads. For example, DNA of wheat inoculated with fungal pathogens was extracted by homogenization using a protocol designed for fungi for long-read sequencing^44^, and at most 5.7% of the total sequence length was identified as the target fungal pathogen by BLASTN^19^. Therefore, the DNA extraction methods used here for *Cps* and boxwood may have the potential to be successful with other fungal plant pathogens as well.

Compared to the detection of bacterial plant pathogens by metagenomic sequencing, fungal plant pathogens present another challenge. Prokaryotic genome databases include dozens, or even hundreds, of genome sequences for most bacterial plant pathogen species, while genome sequences of fungal plant pathogens are still relatively rare in genome databases. This could contribute to the relatively low number of sequencing reads identified as being of fungal origin compared to bacterial origin in some metagenomic studies^25^. In our study, we were unable to use the ONT-provided WIMP taxonomic classification tool for metagenomic analysis when starting this project because *Cps* genomes were not included in the WIMP database. We thus had to build our own custom databases for use with the bioinformatics tools employed here. Fortunately, several genome sequences of *Cps* and *Che* became publicly available by the end of this project and could be included in our large database. Although BLASTN, MetaMaps and Kraken 2 were all adequate in identifying the target plant pathogen using our databases, sensitivity varied. For example, a larger number of *Cps* reads was identified by Kraken 2 compared to BLASTN for most diseased samples, and fewer false-positive reads were identified by Kraken 2 in the negative control. On the other hand, a significant number of reads was assigned by Kraken 2 to non-specific species complexes or genera in the family Nectriaceae.

It is worth noting that *Che*, which is not present in the USA, was identified in diseased samples at very low abundance of 0.001–0.807% by BLASTN (0.000–0.399% by MetaMaps, 0.012–0.312% by Kraken 2). This indicates that all three tools were mostly able to differentiate *Cps* from the closely related species *Che*. Moreover, besides these reads misidentified as *Che*, a small number of reads were identified as *Cps* in the negative healthy control sample. In both cases, when performing BLASTN on these potential false *Che*- and *Cps-*positive reads against the entire NCBI nt database, the best matches for these reads were plants, bacteria, and other fungi. For reads shorter than 100 nt, sometimes *Che* or *Cps* were the best hits but percent identity and bit-score were very low (data not shown). Therefore, the wrongly identified reads were mostly a result of using relatively small custom fungal databases lacking plant, bacteria, and other fungal genomes. We chose to use these relatively small custom databases to accelerate read identification but the resulting false positives are clearly a weakness resulting from this decision. Larger, more comprehensive databases and filtering out short reads can be expected to avoid false positives almost completely. However, it may be impossible to avoid all misidentifications since some reads may get misidentified because they align to genes highly conserved within the genus or family of interest.

It was expected that reads of the Volutella pathogens *P. foliicola* and *P. buxi* would be identified in all diseased samples since they are ubiquitous boxwood pathogens. However, it was interesting that not a single read of either pathogen was identified in the two healthy negative control samples, suggesting that these pathogens only thrive in co-infection with *Cps*. It was also expected that very few reads of *F. graminearum* would be recovered because this species does not cause disease on boxwood. Also, prokaryotes were identified in all samples as described in Supplementary Results 1.

Besides distinguishing between species, metagenomics was shown to almost reach strain/lineage-level precision for plant pathogenic bacteria^23^. *Cps* has diversified into multiple lineages with several of them being present in the US^43,45^. Neither MetaMaps nor Kraken 2 can easily distinguish between lineages since they rely on NCBI taxIDs and only a single taxID is associated with each fungal species. Also, MinION reads have a relatively high error rate and Illumina reads are short, further complicating precise identification. However, we have shown here that assembling MinION reads made it possible to determine which public *Cps* genome sequences were most similar to the *Cps* sequences in some of our samples using either BLASTN or sourmash. Both tools identified the same three strains as best hits, including the strains CBS139395 and CBS139394, both isolated from sweet box (*Sarcococca* spp.) in the same location in Maryland, USA^10^, and both members of clade B^43^. While this result is not sufficient to conclude that the *Cps* strain from our Virginia samples belongs to the same clade, it shows the potential of metagenomic sequencing to reach strain/lineage-level resolution not only for bacteria but also for fungi. Using the obtained *Cps* genome assembly as input into a single nucleotide polymorphism (SNP) pipeline for phylogenetic tree construction will be necessary to confidently assign it to clade B. Also, sequencing a sample on an entire flow cell should provide a higher number of *Cps* reads to obtain a better genome assembly compared to the one we were able to obtain, which had a limited number of complete genes.

Compared to Illumina sequencing, the MinION revealed several strengths. First, the requirements of DNA quantity and quality were lower. Second, with long reads, initial identification using the MinION can be made without assembling metagenomes. Also, its portability and ability to report results in real-time can’t be matched by Illumina. Although the relatively high error rate of the MinION is often considered a weakness, it was not a limitation in our study. The increased length of reads compared to Illumina provided high confidence read identification and easily compensated for the higher error rate.

With regard to detection, 200 MinION reads would have been sufficient to consistently detect *Cps* in the samples with the highest percentage of *Cps* reads. The MinION was also able to detect *Cps* in moderately diseased boxwood, although the percentage of reads identified was lower than 1% and, therefore, a much higher number of reads would be required to confidently detect *Cps*. We did not have the opportunity to determine the detection limit for infected but asymptomatic boxwood. Moreover, infection severity may vary significantly between different asymptomatic samples and it may thus be challenging to determine how many reads would be required without finding *Cps* to confidently conclude that *Cps* is absent. On the other hand, the very low false positive rate provides confidence in identifying an infection even when a very small number of *Cps* reads were detected. Since we had no access to *Cps*-specific molecular PCR or LAMP assays, we cannot compare detection sensitivity of metagenomic sequencing using the MinION with these assays and can only generally state that the sensitivity of metagenomic sequencing increases with the number of total sequencing reads that are generated. Therefore, if high sensitivity of detection is required, one can increase the total number of reads by using an entire flow cell per sample or even using more than one flow cell.

A current challenge with metagenomic sequencing for pathogen identification is that knowledge of bioinformatics is required when using many of the open-source tools designed for this purpose. Although the BLAST program can be performed locally, for higher speed and efficiency, it had to be installed on Virginia Tech’s high performance computer network, ARC. To automate the comparison of every individual sequencing read to our databases and to summarize the obtained results, custom scripts needed to be written. Also, MetaMaps, Kraken 2, and sourmash were run on ARC because the amount of sequence data obtained in metagenomics is too much to handle for a standard laptop or desktop computer. This is an obvious challenge when trying to implement metagenomics into routine disease diagnostics. A user-friendly program interface and automated pipelines running at the back-end on a high-performance computing network will both be required. If these become available, a diagnostic clinic could extract DNA from a sample, prepare a sequencing library, and start a sequencing run within hours and obtain first results on the same day. This would represent a significant acceleration compared to any culture-dependent diagnostic technique and even applicable to the detection of emerging pathogens for which no specific qPCR test may be available.

In conclusion, we have shown here that using appropriate DNA extraction techniques and bioinformatics tools and genome databases, metagenomic sequencing using the ONT MinION can easily distinguish the boxwood blight pathogens *Cps* and *Che* from each other and from other fungal species. With some improvements to databases and parameters used in the classification pipeline, it should be possible to eliminate false positives to practically zero. Using a high enough number of reads, metagenomic sequencing with the ONT Minion can also reach very high sensitivity of detection and specificity can approach strain-level resolution. The main challenge to implementing metagenomic sequencing for plant pathogen identification in routine diagnostics will be in providing access to high performance computing networks and user-friendly interfaces from which to run the necessary computational pipelines.

## Supplementary Information

Supplementary Information.

## Data Availability

Sequencing data have been submitted to the NCBI SRA database under BioProject PRJNA750039, BioSamples SAMN20428190 to SAMN20428209 and SRA Accession numbers SRR15275531 to SRR15275520.
