# Supplementary material for: Metagenomic sequencing for detection and identification of the boxwood blight pathogen Calonectria pseudonaviculata
Source: Sci Rep. 2022 Jan 26;12:1399. doi: 10.1038/s41598-022-05381-x (PMC8791934; doi:10.1038/s41598-022-05381-x)
Supplement: Supplementary file 1 — Supplementary Information. [file 41598_2022_5381_MOESM1_ESM.pdf]

Metagenomic Sequencing for Detection and Identification of the Boxwood Blight Pathogen *Calonectria pseudonaviculata*

Shu Yang[1], Marcela A. Johnson[1,2], Mary Ann Hansen[1], Elizabeth Bush[1], Song Li[1], Boris A. Vinatzer[1*]

1 School of Plant and Environmental Sciences, Virginia Tech, Blacksburg, VA, United States

2 Graduate Program in Genetics, Bioinformatics, and Computational Biology, Virginia Tech, Blacksburg, VA, United States

Supplementary Materials

Supplementary Figure 1. Diseased boxwood naturally infected with *C. pseudonaviculata*.

Supplementary Figure 2. Krona plots based on the percentage reads classified using the custom database with 29 genomes by Kraken 2.

Supplementary Table 1. List of genomes used in the extensive custom database with BLASTN, Kraken 2, and/or sourmash.

Supplementary Table 2. Fungal hits obtained with BLASTN and Kraken 2.

Supplementary Table 3. The 31 reads of sample S1 that had been identified as *C. henricotiae* when using a custom library were compared against NCBI's entire database using BLASTN and the best hits for each read are shown below. None of the reads was identified as *C. henricotiae* showing that using a larger library would have eliminated the false positives for *C. henricotiae*.

Supplementary Table 4. Sequencing statistics of G10 with ONT MinION and Illumina HiSeq.

Supplementary Results 1. Boxwood infected with *Cps* harbored different prokaryotic communities compared to healthy boxwood.

Supplementary Figures

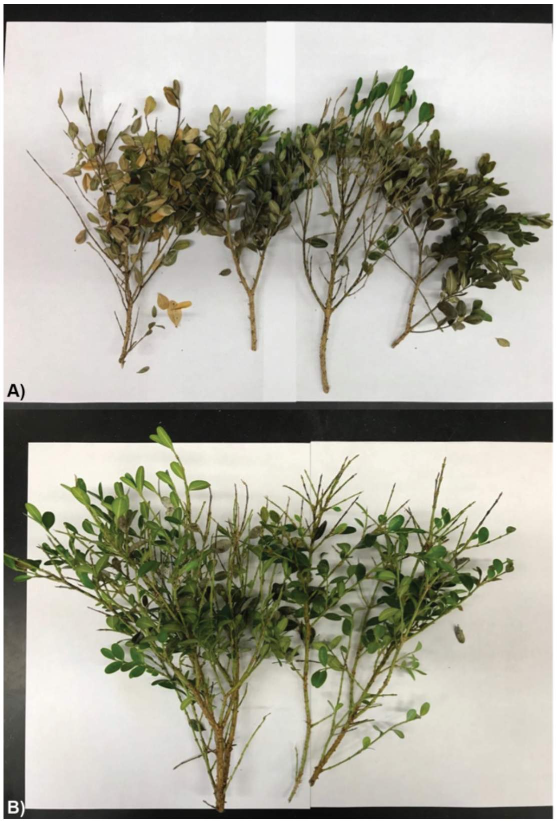

Supplementary Figure 1. Diseased boxwood naturally infected with *Cps*. A) Moderately diseased boxwood. B) Severely diseased boxwood.

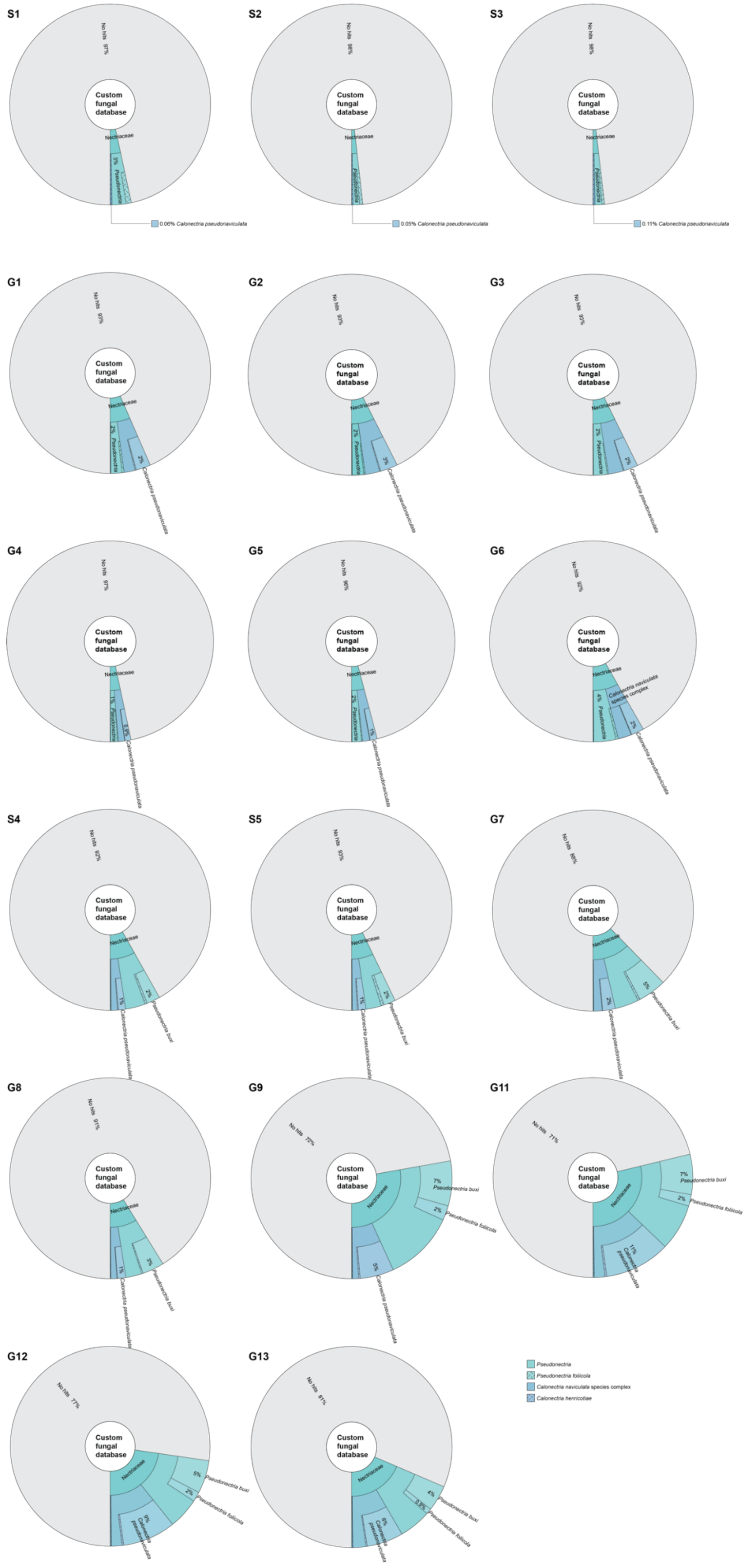
S1
S2
S3
G1
G2
G3
G4
G5
G6
S4
S5
G7
G8
G9
G11
G12
G13
Custom fungal database
Nectriaceae
0.06% Calonectria pseudonaviculata
0.05% Calonectria pseudonaviculata
0.11% Calonectria pseudonaviculata
Pseudonectria
Pseudonectria folliicola
Calonectria naviculata species complex
Calonectria henricotiae

Supplementary Figure 2. Krona plots based on the percentage reads classified using the custom database with 29 genomes by Kraken 2. Each color represents a clade. Each plots represents a sample sequenced by the MinION sequencer.

Supplementary Table S1. List of genomes used in the extensive custom database with BLASTN, Kraken 2, and/or sourmash

Genome	Strain	Accession number
Calonectria henricotiae	CB077	GCA_004380935.1
	CBS138102	GCA_004380885.1
	NL009	GCA_004380965.1
	NL017	GCA_004382205.1
Calonectria pseudonaviculata	CB002	GCA_004141935.1
	CB002	GCA_006505905.1
	CBS139394	GCA_001696505.1
	CBS139395	GCA_004380915.1
	CBS14417	GCA_004381005.1
	CT13	GCA_004380985.1
	ICMP14368	GCA_004382245.1
	NC-BB1	GCA_004381035.1
	ODA1	GCA_004382225.1
Fusarium graminearum	233423	GCA_000966635.1
	241165	GCA_000966645.1
	CS3005	GCA_000599445.1
	DAOM18037	GCA_001717915.1
	FG078	GCA_006942295.1
	FN009	GCA_900476405.1
	ITEM124	GCA_002352725.1
	MDC_Fg1	GCA_900492705.1
	MDC_Fg13	GCA_901446245.1
	NRRL28336	GCA_001717905.1
	PH-1	GCA_900044135.1
	TaB10	GCA_012959185.1
Pseudonectria foliicola	AR2711	GCA_002911195.1
	JAC18-02	GCA_003693505.1
Pseudonectria buxi	AR2414	GCA_003693545.1
	JAC17-19	GCA_003693515.1

Supplementary Table S2. Fungal hits obtained with BLASTN and Kraken 2

Sample ID	# Total reads	**BLASTN**							**Kraken 2**							
		# Hits of *Calonectria henricotiae*	# Hits of *Calonectria pseudonaviculata* (*Cps*)	# Hits of *Fusarium graminearum*	# Hits of *Pseudonectria foliicola*	# Hits of *Pseudonectria buxi*	% Identified reads	% *Cps* hits	# Hits of *Calonectria henricotiae*	# Hits of *Cps*	# Hits of *Fusarium graminearum*	# Hits of *Pseudonectria foliicola*	# Hits of *Pseudonectria buxi*	# Hits of non-specific Nectriaceae	% Identified reads	% *Cps* hits
S1	424,599	31	225	54	8,897	1,203	2.452%	0.053%	320	264	202	1843	1761	10022	3.394%	0.062%
S2	469,952	10	188	36	5,312	763	1.342%	0.040%	426	234	107	1284	1113	5314	1.804%	0.050%
S3	351,180	9	410	18	3,555	471	1.271%	0.117%	366	400	90	802	704	4170	1.860%	0.114%
G1	694,805	91	6,817	58	4,638	513	1.744%	0.981%	913	16,342	316	1,199	1,098	25,572	6.540%	2.352%
G2	1,965,786	219	20,010	69	8,005	598	1.470%	1.018%	3,041	53,424	631	2,411	1,871	81,733	7.280%	2.718%
G3	1,914,124	103	9,117	42	5,777	429	0.808%	0.476%	2,391	42,315	485	2,278	1,712	86,925	7.111%	2.211%
G4	2,644,721	60	5,281	53	4,025	280	0.367%	0.200%	1,561	24,633	480	1,551	1,228	56,420	3.247%	0.931%
G5	3,721,368	113	10,798	86	10,060	691	0.584%	0.290%	2,542	40,843	933	3,400	2,552	98,586	4.000%	1.098%
G6	454,974	38	3,174	21	3,808	269	1.607%	0.698%	450	8,944	231	1,040	719	25,519	8.111%	1.966%
S4	2,358,938	73	4,377	313	28,795	16,572	2.125%	0.186%	3,158	27,282	1,479	8,420	50,680	101,482	8.160%	1.157%
S5	2,755,500	180	11,002	335	51,566	27,900	3.302%	0.399%	4,256	33,325	1,650	13,050	51,947	92,271	7.131%	1.209%
G7	1,343,224	166	13,087	72	17,263	13,392	3.274%	0.974%	1,266	26,970	466	4,729	61,216	70,771	12.315%	2.008%
G8	2,736,033	173	14,276	105	21,025	20,780	2.060%	0.522%	1,903	36,942	889	5,994	95,452	99,711	8.804%	1.350%
G9	295,648	229	15,178	45	34,849	15,430	22.233%	5.134%	617	16,082	244	6,986	21,340	36,876	27.785%	5.440%
G10	541,576	531	49,667	254	38,866	23,074	20.753%	9.171%	1,644	52,677	824	10,321	34,658	44,071	26.625%	9.727%
G11	289,025	297	29,440	104	23,887	13,364	23.213%	10.186%	901	30,723	365	5,359	18,832	26,969	28.769%	10.630%
G12	885,884	719	69,724	396	50,980	30,563	17.201%	7.871%	2,438	76,053	1,153	14,107	47,211	60,102	22.696%	8.585%
G13	277,471	146	12,417	61	9,330	5,516	9.900%	4.475%	560	16,224	244	2,527	11,523	20,397	18.551%	5.847%
NC	831,673	5	137	60	207	21	0.052%	0.016%	103	18	438	44	132	672	0.169%	0.002%

Table S3. The 31 reads of sample S1 that had been identified as *C. henricotiae* when using a custom library were compared against NCBI's entire database using BLASTN and the best hits for each read are shown below. None of the reads was identified as *C. henricotiae* showing that using a larger library would have eliminated the false positives for *C. henricotiae* .

Sequence ID	Accession	Species	Percent identity (%)	Length (bp)	E-value
0d8e0467-c80a-431b-b29c-d72f4d94fa8f	CP017483.1	*Stenotrophomonas* sp.	85.247	3240	0
0ed46597-f80f-402a-87b7-7be3d6bbb8ed	KU668563.1	*Clonostachys rosea*	84.512	1414	0
124c36d8-165a-4c82-9030-36113710ebfe	KF757229.1	*Acremonium chrysogenum*	83.256	2150	0
126aaebc-5f57-478d-8a1b-a30d020613ff	MT447058.1	*Orbiocrella petchii*	78.953	2789	0
1e2fc753-f7fd-4321-8855-6cd0c5ab4b40	NC_043850.1	*Paecilomyces penicillatus*	75.835	1407	7.98E-158
2d363f0a-7175-4b13-a85d-f907435e20f1	CP023323.1	*Cordyceps militaris*	74.481	964	5.44E-90
3262e0b6-f8bc-4093-adf9-d4be35b3d690	KT585676.1	*Lecanicillium saksenae*	79.4	2165	0
3aba7db5-201d-450d-b83c-7709695964dc	NC_043850.1	*Paecilomyces penicillatus*	78.261	2139	0
3e1f7f4a-54e9-463c-9b84-0db5b7956e92	MK213319.1	*Clonostachys rosea*	75.211	2013	0
42b156f6-9ef0-44a5-b9fb-8914fb642537	NC_043850.1	*Paecilomyces penicillatus*	77.275	2275	0
44777610-d88e-4828-8d1d-3ff9b36120e5	KT731105.1	*Nectria cinnabarina*	78.602	2790	0
5316e12b-191e-4e9d-9668-706ec8be082e	XM_024893633.1	*Trichoderma citrinoviride*	75.984	1778	0
6caeeee3-a331-45a9-b101-3c78b57141f9	CP014168.1	*Sphingomonas panacis*	74.589	913	3.93E-79
6efc4b38-515b-4b96-9284-1bebe77298af	LR792747.1	*Metarhizium brunneum*	81.251	4955	0
7568d60a-e985-47c4-ace7-f22e61d6439f	NC_043850.1	*Paecilomyces penicillatus*	81.072	2277	0
863f5f43-ccb0-4c40-ae4c-3cbc1c4915a9	CP052902.1	*Fusarium oxysporum* f. sp. *koae*	72.368	1748	5.78E-107
8b79c6be-8f97-4eb6-85e7-e62975ba0aff	NC_043850.1	*Paecilomyces penicillatus*	80.19	2110	0
950c6db0-18be-4135-9fae-67bc6c6ccabc	XM_025725124.1	*Fusarium venenatum*	88.06	67	7.89E-09
98190aab-298d-4fa4-abc5-18cbe8b6313b	LR792747.1	*Metarhizium brunneum*	81.07	5251	0
a27132f4-0f48-4e33-a1ef-a06746182cae	KU668563.1	*Clonostachys rosea*	80.963	1329	0
a6b89184-2497-48d3-a0ce-e007159b32ac	NC_043850.1	*Paecilomyces penicillatus*	85.686	1537	0
ca4e70c0-6a70-4949-82bc-3bcd08762d93	NC_043850.1	*Paecilomyces penicillatus*	80.244	2293	0
d2ff4fb3-c2b7-43a2-ad13-53389d4a38ef	CP020875.1	*Trichoderma reesei*	77.891	1963	0
d7436683-d95c-421b-8f63-620af96158d2	CP049930.1	*Ustilaginoidea virens*	75.232	1292	9.89E-151
db59b963-441a-4d8e-a100-988235f39aa8	NC_043850.1	*Paecilomyces penicillatus*	82.965	2260	0
de19d969-1acc-4388-8da8-f4519d096555	KP742838.1	*Fusarium mangiferae*	73.01	2249	8.37E-144
e660b8a2-a17b-4f02-abf1-825159431c8f	KU668563.1	*Clonostachys rosea*	84.768	1162	0
f43202f4-32dc-489a-938e-cb48c8f760f8	MT123351.1	*Calonectria ilicicola*	79.068	3153	0

Note that two reads had no hits at all.

Supplementary Table S4. Sequencing statistics of G10 with ONT MinION and Illumina HiSeq

Sample	G10		Negative control	
Platform	Illumina	MinION	Illumina	MinION
# Toatl reads	17,033,700	541,576	271,857,762	831,673
Total read length (bp)	1,498,691,820	1,955,966,076	40,778,664,300	1,945,418,169
Max read length (bp)	100	65,962	150	28,837
Min read length (bp)	2	2	150	4
Avg read length (bp)	88	3,612	150	2,339

Supplementary Results 1

Boxwood infected with *Cps* harbored different prokaryotic communities compared to healthy boxwood

Methods

A database of all NCBI RefSeq genomes in April 2021 was built to profile boxwood-associated prokaryotic communities using Kraken 2 v2.1.1 [1]. Pavian v1.0 [2] was used to visualize the taxonomic classification of the prokaryotic sequences.

Results

Since metagenomics provides a complete picture of all microbes present in a sample, we decided to also take a look at the bacterial communities associated with boxwood. Therefore, Kraken 2 analysis was performed against a database containing all assembled bacterial genomes in RefSeq. MinION reads were combined based on disease severity and DNA extraction method, forming five composite samples: moderately diseased boxwood extracted after sonication, moderately diseased boxwood extracted by grinding, severely diseased boxwood extracted after sonication, severely diseased boxwood extracted by grinding, and healthy boxwood extracted by grinding. Supplementary Figure 3 shows the taxonomic profile of each group.

Because our DNA samples only came from a small number of boxwood samples, this analysis is necessarily descriptive and preliminary. However, some interesting trends were observed. Proteobacteria was the phylum with the highest relative abundance for all five composite samples (Supplementary Figure 3). Only for healthy

boxwood, Firmicutes was the second most abundant phylum (Supplementary Figure 3E). Another interesting feature of the healthy boxwood sample was the relatively high abundance of *Pasteurella multocida* followed by *Vibrio anguillarum* as the second most abundant species.

The only common feature among all symptomatic samples and absent from the healthy plant sample was the presence of *Pseudomonas putida* and *Pseudomonas fulva*. Bacterial communities of the severely diseased samples also included a relatively high abundance of *Pseudomonas monteilii* and *Pseudomonas rizosphereae*. While *Stenotrophomonas maltophilia* and other *Stenotrophomonas* species were relatively abundant in moderately diseased boxwood, the genera *Pantoea* and *Erwinia* were of higher relative abundance in severely diseased boxwood. Some bacterial species, such as *Buchnera aphidicola,* may have been present due to insects or due to human handling, (*Massilia oculi)*.

Discussion

Although these results are preliminary because of the small number of samples that were examined, the prokaryotic community composition appears to differ between healthy and diseased boxwood. The role of the detected bacterial species in regard to its effect on disease severity will need to be determined. Also, if some boxwood-associated community members may possibly have disease-suppressive ability that could be leveraged for disease management, similar to bacteria previously isolated from recycling irrigation systems [3], should be determined.

References

1 Wood, D. E., Lu, J. & Langmead, B. Improved metagenomic analysis with Kraken 2. *Genome Biol* **20**, 257, doi:10.1186/s13059-019-1891-0 (2019).

2 Breitwieser, F. P. & Salzberg, S. L. Pavian: interactive analysis of metagenomics data for microbiome studies and pathogen identification. *Bioinformatics* **36**, 1303-1304, doi:10.1093/bioinformatics/btz715 (2020).

3 Yang, X. & Hong, C. Biological control of boxwood blight by *Pseudomonas protegens* recovered from recycling irrigation systems. *Biological Control* **124**, 68-73, doi:https://doi.org/10.1016/j.biocontrol.2018.01.014 (2018).

Supplementary Figure 3. Sankey plots for taxonomic profiling of the metagenome based on reads mapped against RefSeq complete bacterial genomes. The heights of the rectangles indicate the number of reads assigned per taxa and rank, also indicated above/next to each taxa. A) Results of all moderately diseased samples (S1 – S3) that were sonicated. B) Results of all moderately diseased samples (G1 – G6) that were homogenized in liquid nitrogen. C) Results of all severely diseased samples (S4 – S5) that were sonicated. D) Results of all severely diseased samples (G7 – G13) that were homogenized in liquid nitrogen. E) Results of the negative control (NC).

See next page for Figure.

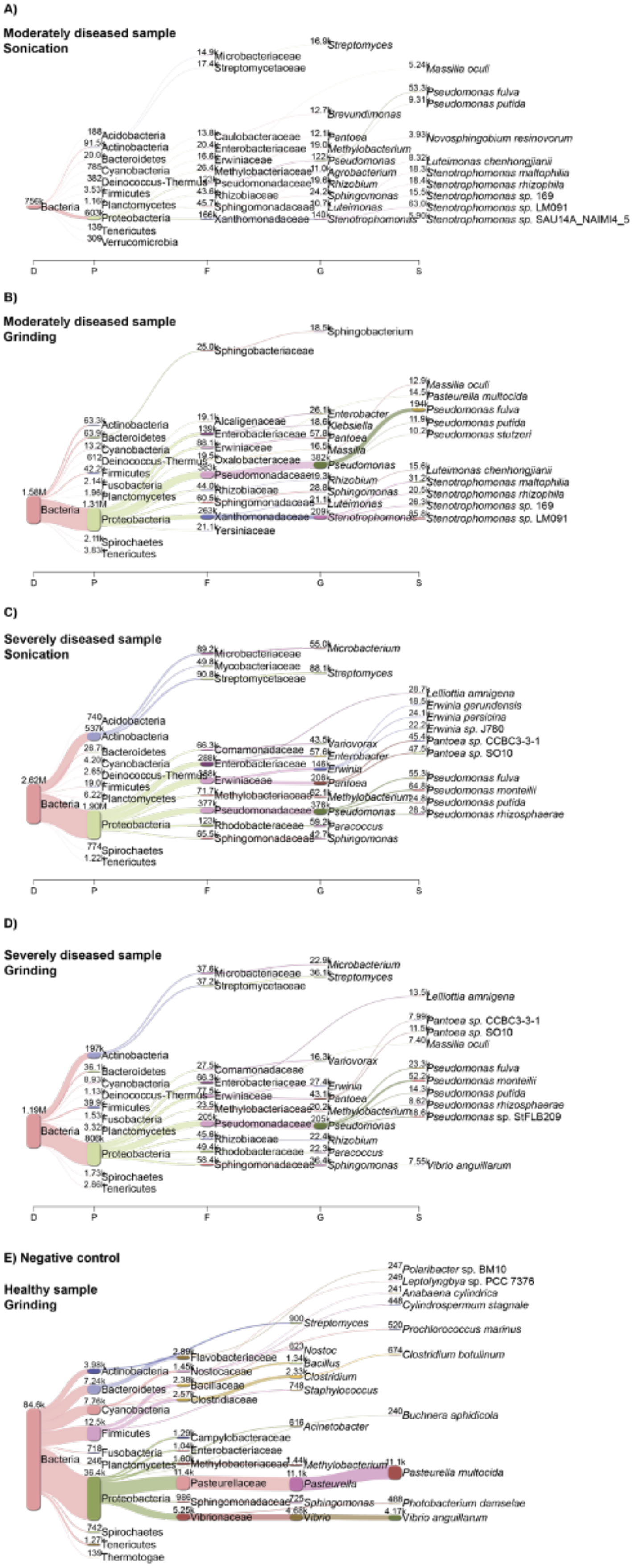
A)
Moderately diseased sample
Sonication
B)
Moderately diseased sample
Grinding
C)
Severely diseased sample
Sonication
D)
Severely diseased sample
Grinding
E) Negative control
Healthy sample
Grinding
